# Hospitalization and Antimicrobial Resistance in *Salmonella* Outbreaks, 1984–2002

**DOI:** 10.3201/eid1106.041231

**Published:** 2005-06

**Authors:** Jay K. Varma, Katherine D. Greene, Jessa Ovitt, Timothy J. Barrett, Felicita Medalla, Frederick J. Angulo

**Affiliations:** * Centers for Disease Control and Prevention, Atlanta, GA, USA;; † Private practice, Lenoir, NC, USA

**Keywords:** salmonella, outbreaks, hospitalization, antibiotic resistance

## Abstract

Few studies have evaluated the health consequences of antimicrobial-resistant *Salmonella* strains associated with outbreaks. Among 32 outbreaks occurring in the United States from 1984 to 2002, 22% of 13,286 persons in 10 *Salmonella*-resistant outbreaks were hospitalized, compared with 8% of 2,194 persons in 22 outbreaks caused by pansusceptible *Salmonella* strains (p<0.01).

Nontyphoidal *Salmonella* strains are a frequent cause of foodborne disease outbreaks in the United States; they account for ≈13% of outbreaks reported to the Centers for Disease Control and Prevention (CDC) from 1993 to 1997 (1). Antimicrobial resistance is common among salmonellae and has been increasing, particularly in *Salmonella enterica* serotype Typhimurium, the most common *Salmonella* serotype (2,3). In the 1990s, a strain of *S*. Typhimurium resistant to ampicillin, chloramphenicol, streptomycin, sulfonamides, and tetracycline (R-type ACSSuT) emerged in the United States and Europe; most of these isolates were phage definitive type 104 (DT104) (2).

Antimicrobial therapy is not required for most *Salmonella* infections, but it may be lifesaving in patients with or at risk for extraintestinal infection (4). Increasing levels of antimicrobial resistance are concerning because treatment may fail if the infecting strain of *Salmonella* is resistant to the prescribed agent (5). Also, when the proportion of *Salmonella* strains that are resistant increases, the total prevalence of human *Salmonella* infections increases (6). Resistant salmonellae preferentially cause illness in persons who take antimicrobial drugs for medical conditions unrelated to *Salmonella* infection (7–9).

One previous study has formally evaluated the human health consequences of antimicrobial-resistant *Salmonella* strains associated with outbreaks. In 1984, Holmberg et al. reviewed CDC investigations conducted from 1973 to 1983 to determine the rate of hospitalization and death in outbreaks caused by antimicrobial-resistant salmonellae compared with outbreaks caused by pansusceptible salmonellae (10). Because the epidemiology of both *Salmonella* infections and antimicrobial resistance has changed in the past 20 years, we repeated this analysis for outbreaks investigated from 1984 to 2002.

## The Study

We reviewed final reports of nontyphoidal *Salmonella* outbreaks investigated by CDC from 1984 to 2002. We excluded outbreaks that occurred in a healthcare setting or outside the United States, including on cruise ships. When antimicrobial susceptibility was not recorded in the final report, we searched CDC microbiology records for susceptibility test results on isolates collected as part of the outbreak. Outbreaks were only included if susceptibility data were available for >1 isolate. Because different laboratories performed susceptibility testing, the antimicrobial agents tested and the methods used varied between outbreaks. We classified outbreaks as resistant when the outbreak strain was resistant to ≥1 antimicrobial agent; other outbreaks were considered pansusceptible. Outbreaks caused by resistant strains were additionally classified as R-type AC/KSSuT when the outbreak strain was at least resistant to ampicillin, chloramphenicol or kanamycin, streptomycin, sulfamethoxazole, and tetracycline. Outbreaks were additionally classified as resistant to a clinically important agent when the outbreak strain was at least resistant to ampicillin, trimethoprim-sulfamethoxazole, aminoglycosides, fluoroquinolones, or a third-generation cephalosporin.

Data from the final investigative reports were used for the analysis. When analyzing data according to outbreaks, we calculated the medians for percentage hospitalized and percentage who died and compared medians with the Wilcoxon rank-sum test. When analyzing data according to ill persons, we pooled data from the reports, calculated proportions, and compared proportions with chi-square or, when appropriate, Fisher exact test. The denominators for percentage hospitalized and died varied depending on the number of persons in whom outcome data were ascertained. All p values were 2-tailed. Data were analyzed by using SAS v.9 (SAS Institute, Cary, NC, USA).

From 1984 to 2002, CDC investigated 48 community outbreaks of nontyphoidal *Salmonella* strains in the United States. Of these, 47 (98%) had a final report available for review (Table A1). We restricted our analyses to the 39 (83%) outbreaks in which data about antimicrobial susceptibility were available. These 39 outbreaks affected 23,206 persons. The largest outbreak occurred in 1985, in which culture-confirmed *S*. Typhimurium infection associated with milk consumption developed in 16,659 persons (11).

Strains from 11 (28%) outbreaks were resistant, and 28 (72%) were pansusceptible. The 11 outbreaks caused by resistant strains involved 18,698 persons. Of these 11 outbreaks, 7 (64%), involving 17,182 persons, had strains that were at least R-type AC/KSSuT, and 9 (82%), involving 17,919 persons, had strains that were resistant to a clinically important agent.

Hospitalization data were available for 32 outbreaks involving 21,702 ill persons. The hospitalization rates were higher for each type of outbreak caused by resistant salmonellae compared with outbreaks caused by susceptible salmonellae (p<0.01 for each comparison) (Table). To account for differences in the size of outbreaks, we compared the median proportion of persons hospitalized and compared hospitalization rates after excluding a large *Salmonella* outbreak that occurred in 1985. The median proportion hospitalized for each type of outbreak caused by resistant strains (26%) was >2.5 times higher than the median proportion hospitalized for outbreaks caused by pansusceptible strains (10%, p<0.05 for all resistance patterns). The difference in hospitalization rates between outbreaks caused by resistant and susceptible strains was similar after we excluded the large 1985 outbreak of resistant *S*. Typhimurium, in which the percentage hospitalized was 22%. The results also remained similar after excluding *S*. Enteritidis outbreaks, in which rates of hospitalization and isolate resistance were low.

**Table:** Hospitalization and death rates among nontyphoidal *Salmonella* outbreaks by resistance pattern, 1984–2002*

Resistance pattern	Outbreaks		Patients	
	n (median % [range])	p value	No./total (%)	p value
Hospitalization				
Pansusceptible	22 (9.7 [0–37.5])	Referent	164/2,194 (7.5)	Referent
Resistant >1	10 (26.2 [9.3–49.3])	<0.01	2,913/13,286 (21.9)	<0.01
R-type AC/KSSuT	7 (26.1 [9.3–48.9])	0.02	2,827/12,806 (22.1)	<0.01
Clinically important agent	9 (26.1 [9.3–48.9])	0.04	2,877/13,213 (21.8)	<0.01
Death				
Pansusceptible	16 (0 [0–0.6])	Referent	2/3,283 (0.06)	Referent
Resistant >1	8 (0.1 [0–1.4])	0.05	23/18,644 (0.1)	0.57
R-type AC/KSSuT	5 (0 [0–0.7])	0.21	20/17,150 (0.1)	0.56
Clinically important agent	6 (0 [0–0.7])	0.69	20/17,865 (0.1)	0.80

*AC/KSSut, ampicillin, chloramphenicol, kanamycin, streptomycin, sulfamethoxazole, tetracycline.

Mortality data were available for 24 outbreaks involving 21,927 persons. A greater proportion of persons died in resistant outbreaks than in pansusceptible outbreaks, but the difference was not significant (0.1% in outbreaks caused by resistant strains vs. 0.06% in outbreaks caused by pansusceptible strains, p = 0.57) (Table).

The 8 outbreaks in which no susceptibility data were available involved 1,914 ill persons. Three (38%) outbreaks were due to *S*. Enteritidis and 2 (25%) to *S*. Typhimurium. In the 6 outbreaks for which hospitalization data were available, 70 (20%) of 353 persons were hospitalized. In the 4 outbreaks for which mortality data were available, 7 (0.4%) of 1,708 persons died.

## Conclusions

Outbreaks caused by antimicrobial-resistant, nontyphoidal salmonellae were associated with an increased rate of hospitalization compared with outbreaks caused by pansusceptible salmonellae. The results were similar regardless of the definition of resistance used. This association has been found previously in studies of sporadic illness (12,13).

Several possible mechanisms may explain the higher hospitalization rate. Persons who take antimicrobial drugs for reasons unrelated to gastroenteritis have an increased risk of developing antimicrobial-resistant *Salmonella* infections; such patients may be taking antimicrobial drugs because they have medical conditions that increase their risk for hospitalization (7–9). We doubt that this explains the differential hospitalization rate observed in our study, because the outbreaks we studied occurred in diverse community settings. A second explanation for the higher hospitalization rate is that persons with resistant *Salmonella* infections may fail empiric antimicrobial treatment, and their physicians subsequently hospitalize them for inpatient therapy. A third explanation is that resistant salmonellae may be more virulent because of some unknown factor. In the United States, resistant salmonellae are more often associated with hospitalization and bloodstream infection compared to pansusceptible salmonellae (13). In Canada and Denmark, studies have also found excess death rates associated with resistant *Salmonella* infection (14,15). In England and Wales, a study found no association between resistance and bloodstream infection, but that study had substantial limitations, including the failure to use pansusceptible salmonellae as a referent group and the failure to adjust for confounders, such as age (16).

Since the review of *Salmonella* outbreaks published in 1984, several changes have occurred in the epidemiology of antimicrobial-resistant salmonellae. First, *S*. Typhimurium DT104 has emerged as a cause of antimicrobial-resistant *Salmonella* infections. Five outbreaks in this study were caused by *S*. Typhimurium with R-type AC/KSSuT; isolates with this resistance pattern are often DT104 (2,17). The hospitalization rate was higher in these 5 outbreaks (median proportion hospitalized: 17%) than in 3 outbreaks caused by pansusceptible *S*. Typhimurium, which suggests that resistance or related factors, rather than just serotype, contribute to the differential rates of hospitalization. Second, a strain of *S*. Newport resistant to at least 9 agents and with diminished susceptibility to ceftriaxone recently emerged in the United States; 1 outbreak in this review was caused by this strain and had a hospitalization rate greater than that seen in most other resistant outbreaks (18). Third, *S*. Enteritidis has emerged as a frequent cause of foodborne outbreaks (19). *S*. Enteritidis strains are infrequently resistant to antimicrobial agents, and in this review, *S*. Enteritidis outbreaks were associated with low hospitalization rates. Excluding *S*. Enteritidis outbreaks did not change the results of our analysis.

The previous analysis by Holmberg et al. found that the rate of death was significantly greater in outbreaks caused by resistant strains (4%) compared with outbreaks caused by pansusceptible strains (0.2%) (10). Rates of death in our study were lower. Because this study covered a more recent period, improvements in medical care may explain the overall lower death rates. Studies with larger sample sizes are needed to determine whether antimicrobial resistance is associated with increased fatality rates.

Our study has some limitations, the most important of which is selection bias. State health departments investigate hundreds of *Salmonella* outbreaks annually. Occasionally, state health departments request assistance from CDC in investigating these outbreaks. The reasons for such requests vary but may be related to size, severity, setting, or other features of the outbreak. While concern about a high hospitalization rate might encourage a state health department to request assistance from CDC in a *Salmonella* outbreak, susceptibility results are usually not known initially and are unlikely by themselves to influence the request for assistance.

Another limitation is that our study was based on final reports, not raw data. As a result, we were unable to adjust for the different patient populations affected, including age, medical condition, and other factors that could affect hospitalization. Excluding outbreaks that occurred in healthcare settings may have reduced some of this bias. Adjustment for serotype, which could also affect hospitalization rates, was also not possible because of the small sample size.

Despite these limitations, we believe this study adds to the weight of evidence about the health effects of antimicrobial-resistant salmonellae. The evidence now includes data from outbreaks and sporadic illness in the United States from 1970 to 2002. Data from sporadic illness and 1 outbreak is also available from Denmark (14,20). Across these diverse studies, higher hospitalization rates are consistently found in patients infected with resistant salmonellae. Such data about the adverse human health effects of resistant salmonellae should be incorporated into programs that promote appropriate antimicrobial use in humans and animals.

## Appendix

**Table A1:** Domestic, community, nontyphoidal *Salmonella* outbreaks investigated by the Centers for Disease Control and Prevention, 1984–2002

Year	State	Serotype	Resistance pattern*	Implicated vehicle	No. hospitalized/total (%)†	No. died/total (%)‡
1984	Oregon	Typhimurium	TS	Salad bar	45/388 (12)	0/715 (0)
1984	Pennsylvania	Infantis	Pansusceptible	Turkey/beef sandwich	1/203 (0)	0/203 (0)
1985	California	Newport	ACKSSuT	Ground beef	22/45 (49)	2/298 (1)
1985	Missouri	Thompson	NA	Pork	NA	NA
1985	Illinois	Typhimurium	AKSSuT	Milk	2777/12624 (22)	18/16659 (0)
1986	Georgia	Typhimurium	Pansusceptible	Turkey	4/719 (1)	0/719
1986	Multiple§	Enteritidis, Heidelberg	Pansusceptible	Eggs (in frozen pasta)	13/166 (8)	1/166 (1)
1987	Georgia	Havana	T	Chicken	36/73 (49)	1/73 (1)
1987	Hawaii	Agona	Pansusceptible	Chicken	NA	0/202 (0)
1989	Maine	Heidelberg	Pansusceptible	Unknown	NA	NA
1989	Minnesota	Javiana	Pansusceptible	Cheese	NA	NA
1989	Multiple§	Chester	Pansusceptible	Cantaloupe	NA	NA
1989	Nevada	Typhimurium	Pansusceptible	Deli platter	NA	NA
1990	Pennsylvania	Enteritidis	Pansusceptible	Eggs	5/26 (19)	0/94 (0)
1990	South Carolina	Agona	NA	Turkey	NA	0/850 (0)
1992	Georgia	Infantis	Pansusceptible	Eggs (in rice pudding)	0/113 (0)	0/113 (0)
1992	New York	Enteritidis	NA	Eggs (in mayonnaise)	8/98 (8)	0/98 (0)
1993	Missouri	Typhimurium	NA	Water	13/27 (48)	7/650 (1)
1993	Multiple§	Montevideo	Pansusceptible	Raw tomatoes, lettuce	15/62 (24)	0/84 (0)
1993	Texas	Enteritidis	NA	Egg rolls	2/19 (11)	NA
1994	Georgia	Enteritidis	Pansusceptible	Eggs (in ice cream)	0/129 (0)	NA
1995	Arizona	Stanley	KTB	Alfalfa sprouts	5/19 (26)	NA
1995	Florida	Hartford	Pansusceptible	Orange juice	7/32 (22)	NA
1995	Utah	Enteritidis	Pansusceptible	Eggs	5/43 (12)	NA
1996	Colorado	Enteritidis	Pansusceptible	Komodo dragon	8/37 (22)	NA
1996	South Dakota	Thompson	Pansusceptible	Roast beef	0/43 (0)	0/52 (0)
1996	Hawaii	Enteritidis	Pansusceptible	Unknown	NA	NA
1997	Washington	Typhimurium	ACSSuT	Cheese (queso fresco)	5/54 (9)	0/54 (0)
1997	Vermont	Typhimurium	ACSSuT	Milk (raw)	1/9 (11)	NA
1997	Maryland	Heidelberg	KST	Ham (stuffed)	NA	2/706 (0)
1997	Kansas, Missouri	Infantis, Anatum	Pansusceptible	Sprouts	18/67 (27)	0/114 (0)
1997	South Carolina	Enteritidis	Pansusceptible	Eggs	4/192 (2)	0/192 (0)
1997	Nevada	Enteritidis	Pansusceptible	Eggs (in hollandaise sauce)	15/63 (24)	0/91 (0)
1998	Ohio	Typhimurium	AKSSuT	Farm exposure	5/19 (26)	NA
1998	Multiple§	Agona	Pansusceptible	Oat cereal	33/88 (38)	1/409 (0)
1998	Massachusetts	Typhimurium	Pansusceptible	Milk (raw)	2/47 (4)	NA
1999	Colorado	Typhimurium	Pansusceptible	Clover sprouts	3/20 (15)	0/81 (0)
1999	Massachusetts	Newport	MDR-AmpC	Farm exposure	11/32 (34)	0/46 (0)
1999	Missouri	Typhimurium	NA	Water	17/110 (15)	0/110 (0)
2000	Multiple§	Bareilly	NA	Water	19/46 (41)	NA
2000	Multiple§	Enteritidis	Pansusceptible	Orange juice	13/88 (15)	NA
2000	North Carolina	Enteritidis	NA	Eggs	11/53 (21)	NA
2000	Pennsylvania, New Jersey	Typhimurium	AKSSuT	Milk	6/23 (26)	0/93 (0)
2001	Mississippi	Javiana	Pansusceptible	Amphibian exposure	9/57 (16)	0/73 (0)
2002	Florida	Javiana	Pansusceptible	Tomatoes	3/82 (4)	NA
2002	Texas	Enteritidis	Pansusceptible	Unknown	3/41 (7)	0/650 (0)
2002	Washington, DC	Muenchen	Pansusceptible	Pasta salad	3/40 (8)	0/40 (0)

*A, ampicillin; C, chloramphenicol; K, kanamycin; S, streptomycin; Su, sulfamethoxazole; T, tetracycline; B, trimethoprim-sulfamethoxazole; MDR-AMPC, ACSSuT + amoxicillin/clavulanic acid, cephalothin, cefoxitin, ceftiofur, ceftriaxone (reduced susceptibility); NA, not available. †Denominator includes those for whom hospitalization status was ascertained. In many outbreaks, hospitalization was only ascertained for patients enrolled in an analytic study. ‡Denominator includes those for whom mortality was assessed. If this number was not available, the total number of ill persons in the outbreak was included as the denominator. §Outbreak involving ≥3 states.
